# Comparison of Analytical Approaches Predicting the Compressive Strength of Fibre Reinforced Polymers

**DOI:** 10.3390/ma11122517

**Published:** 2018-12-11

**Authors:** Christian Leopold, Sergej Harder, Timo Philipkowski, Wilfried V. Liebig, Bodo Fiedler

**Affiliations:** 1 Institute of Polymer and Composites, Hamburg University of Technology (TUHH), Denickestrasse 15, D-21073 Hamburg, Germany; sergej.harder@tuhh.de (S.H.); tphilipkowski91@web.de (T.P.); fiedler@tuhh.de (B.F.); 2 Institute of Vehicle System Technology, Karlsruhe Institute of Technology (KIT), Rintheimer Querallee 2, D-76131 Karlsruhe, Germany; wilfried.liebig@kit.edu

**Keywords:** fibre reinforced polymer, compression, analytical models, prediction, shear properties, microbuckling, kinking, glass fibres, carbon fibres

## Abstract

Common analytical models to predict the unidirectional compressive strength of fibre reinforced polymers are analysed in terms of their accuracy. Several tests were performed to determine parameters for the models and the compressive strength of carbon fibre reinforced polymer (CFRP) and glass fibre reinforced polymer (GFRP). The analytical models are validated for composites with glass and carbon fibres by using the same epoxy matrix system in order to examine whether different fibre types are taken into account. The variation in fibre diameter is smaller for CFRP. The experimental results show that CFRP has about 50% higher compressive strength than GFRP. The models exhibit significantly different results. In general, the analytical models are more precise for CFRP. Only one fibre kinking model’s prediction is in good agreement with the experimental results. This is in contrast to previous findings, where a combined modes model achieves the best prediction accuracy. However, in the original form, the combined modes model is not able to predict the compressive strength for GFRP and was adapted to address this issue. The fibre volume fraction is found to determine the dominating failure mechanisms under compression and thus has a high influence on the prediction accuracy of the various models.

## 1. Introduction

Fibre reinforced polymers (FRP) are increasingly used for structural parts in many applications owing to their high density specific strength and stiffness. However, in contrast to their excellent tensile properties, the mechanical properties under compressive loading are significantly inferior. Compressive strength is limited to approximately 70% of the tensile strength [1]. Although the macroscopic failure behaviour of FRP under compressive loading is brittle, the failure process is complex and the compressive strength is difficult to predict, leading often to high safety margins. Accurate prediction methods for an optimum design of composite parts with regard to lightweight applications are necessary and different approaches for predicting the compressive strength have been developed.

A first model for predicting compressive strength of composite laminates was presented by Rosen [2]. He proposed that compressive failure initiates due to fibre microbuckling and distinguished between two modes of microbuckling: in-phase microbuckling (shear mode) for higher and out-of-phase microbuckling (extension mode) for lower fibre volume fractions. The in-phase microbuckling leads to the formation of a kink-band with increasing load. Similar to the compressive failure process of wood [3,4], three stages of kinking are distinguished for carbon fibre reinforced polymer (CFRP) [4,5]. Incipient kinking as the first stage initiates on a small scale by localised plastic shearing and buckling of fibres. The localised incipient kinking areas grow and coalesce in the following transient kinking stage to form a single dominant kink-band across the specimen. During the last stage of steady state kinking, the kink-band broadens laterally until final failure [3,4,5]. In addition to the kink band broadening observed experimentally in [4,5], during which a uniform kink band grows in the direction of loading under increasing deformation and at constant stress, Budiansky et al. [6] proposed the second mechanism of transverse kink propagation. Here, the kink band grows transversely to loading direction under constant overall shortening [6]. The initiation of microbuckling and the following kink-band can be described as shear driven fibre failure [7,8]. Thus, the shear strength of the matrix supporting the fibres plays a major role in failure initiation under compression [9].

After damage initiates, delamination, kink-band development and the interaction between these two mechanisms dominate the damage process [10]. In laminates with different fibre orientations, the failure mode is determined by number and orientation of the different layers, the matrix shear properties and the interlaminar fracture properties [10]. In particular, the mode II shear strength has a significant influence on the composite compressive strength and it depends on the interfacial fracture properties, whether a splitting or kinking type of failure occurs [11]. Furthermore, the position of the 0°-layers is critical for stability because, at free edges, first fibre shear failure occurs in a 0°-layer and then leads to local microbuckling and the formation and growth of a kink-band as final failure mechanisms [12]. A detailed overview of the compressive failure of FRP is given by Schultheisz and Waas [13,14] and in a recent review by Soutis [15].

An analytical model for describing fibre microbuckling was developed by Berbinau et al. [16]. In this model, the orientation of the fibres with regard to loading direction is critical [17]. The analytical model of Argon [18] that assumes rigid-plastic microbuckling and the extension from Budiansky [19,20,21], which considers elastic-plastic microbuckling, describe the initiation and propagation of a kink-band with an orientation angle β, the kink-band width, and the inclination angle of the fibres. Initiation of fibre microbuckling and a kink-band is facilitated at defects, e.g., local fibre misalignment [22,23,24,25] or voids [26,27] and can be described for the latter case with an analytical model [28]. It was shown by Bazhenov et al. [29] that the fibre diameter is relevant for compressive strength, especially if flaws such as voids are present. An increasing fibre diameter results in a decreasing flaw sensitivity and hence increased compressive strength compared to specimens with smaller fibre diameter [29]. For application of the plastic kinking and microbuckling analysis, the shear yield properties of the matrix are needed. They can be determined in tensile tests with angle ply laminates via the graphical method from Batdorf and Ko [30,31].

A comparison of the applicability of the fibre kinking and microbuckling model for CFRP under the influence of temperature and moisture showed that the shear strength and fibre imperfections are correctly identified as critical parameters, but the accuracy for compressive strength is limited [9]. Jumahat et al. [32] compared the models from Budiansky [19,20] and Berbinau [16] with experimental results for CFRP and proposed a combined modes model that can be applied by using a graphical method [30,31,32,33]. The unidirectional (UD) compressive strength of FRP is defined as the failure strength under compression loading parallel to the fibre direction. The different analytical models for predicting the UD compressive strength are summed up shortly in Section 2 and then analysed in terms of predictive accuracy and applicability.

The analytical models for compressive strength prediction presented above are designed to be valid for all fibre types and especially for the two most commonly used composites, namely CFRP and glass fibre reinforced polymer (GFRP). However, they are verified only for one of these two materials, mostly for CFRP. Nonetheless, GFRP is also increasingly used in many industries and thus the applicability of the models for composites based on this fibre type is relevant. The aim is thus to validate these common analytical models in terms of predictive accuracy for both CFRP and GFRP. Hereby, the influence of fibre type with regard to diameter and mechanical properties is analysed. A lower fibre volume fraction compared to existing literature is obtained, and the influence of fibre content on the prediction accuracy is discussed as well. Applying the analytical models developed for CFRP also to GFRP is a novelty and it is analysed as to whether they function as desired or whether an adaption is necessary to consider the geometry and mechanical properties of glass fibres. The results are expected to be of great interest for all those who do design or research for GFRP composite laminates.

## 2. Analytical Models for Compressive Strength Prediction of FRP

For predicting the compressive strength $\sigma_{c}$ with the model from Rosen [2], a high enough fibre volume content $V_{f}$ for the shear mode to be applicable is assumed. For this case, the UD compressive strength can be predicted with Equation (1), where $G_{m}$ is the shear modulus of the matrix [2]:

(1) $$\sigma_{c}=\frac{G_{m}}{1-V_{f}}.$$

The fibre kinking model after Argon [18] with the extension from Budiansky [19,20] predicts the compressive strength via the plastic deformation of the composite. The model states that the compressive strength is reached at the transition from elastic to plastic material behaviour. This implies, that the compressive strength is dominated by the matrix properties. It is further assumed that the plastic deformation can be described by pure shear after the shear yield strength $\tau_{y}$ is reached. For small kink-band angles β, the compressive strength can be predicted by applying Equation (2) [19,20]:

(2) $$\sigma_{c}=\frac{\tau_{y}}{\phi_{0}+\gamma_{y}}.$$

In this equation, $\phi_{0}$ is the initial maximum angular fibre misalignment (in radian) and $\gamma_{y}$ is the shear yield strain. For larger kink-band angles, the compressive strength can be predicted with Equation (3). Here, $\sigma_{Ty}$ is the plane-strain yield stress in pure transverse tension [6,19,20]:

(3) $$\sigma_{c}=\frac{\tau_{y}\;\cdot \;\sqrt{1+{\left(\frac{\sigma_{Ty}}{\tau_{y}}\right)}^{2}\;\cdot \;\tan^{2}\beta }}{\phi_{0}+\gamma_{y}}.$$

Berbinau et al. [16] developed an analytical model for compressive strength prediction of FRP based on damage initiation by fibre microbuckling. With this model, the criticality of an off-axis ply orientation is discussed [17]. The initial fibre waviness is modelled by a sine function with amplitude $V_{0}$. From the amplitude change due to an external compressive force, represented by a new sine function with amplitude *V* and half wavelength λ, a differential equation for the displacement *v* of the deflected fibre axis is derived. Assuming linear matrix behaviour and hence a constant matrix shear modulus $G_{m}$, the relationship is simplified to the more easily to solve analytical approach given by Equation (4) [16]:

(4) $$\frac{V}{V_{0}}={\left[1-\frac{P}{E_{f}I{\left(\pi /\lambda \right)}^{2}-A_{f}G_{c}}\right]}^{-1},$$

where *P* is the compressive load on a fibre and $G_{c}$ is the composite shear modulus. $E_{f}$, $A_{f}$ and *I* are the elastic modulus, cross-section area, and second modulus of inertia of the fibres, respectively. The load *P* can be correlated to the global stress on the 0°-ply by using Equation (5) [16,17]:

(5) $$P≅\frac{A_{f}}{V_{f}}\sigma_{0}.$$

A graphic representation of $V/V_{0}$ plotted over compressive stress $\sigma_{0}$ exhibits an asymptotic increase of the fibre amplitude. The asymptotic increase defines the failure stress of the UD FRP laminate [17,32].

The models from Budiansky [19,20] and Berbinau et al. [16] are compared to experimental results for CFRP by Jumahat et al. [32]. It was shown that the fibre kinking model from Budiansky overestimates the compressive strength, whereas the fibre microbuckling model from Berbinau et al. results in an underestimation of compressive strength. This is explained with the fact that the microbuckling model predicts the critical stress at which the fibres fail due to microbuckling. However, the stress at final failure of a composite laminate is dominated by both fibre microbuckling and kink-band formation [32]. Based on these two models, a combined modes model was derived by Jumahat et al. [32] that considers also the plastic deformation after fibre failure due to elastic microbuckling. The compressive stress is calculated according to Equation (6) with the predicted strength from the microbuckling model and an additional plastic kinking part described from the fibre kinking model [32]:

(6) $$\sigma_{c}=\sigma_{microbuckling}+\sigma_{kinking}.$$

The additional kinking stress $\sigma_{kinking}$ is calculated with Equation (7) as follows [32]:

(7) $$\sigma_{kinking}=\frac{\tau_{ult}-\tau_{y}}{\left(\gamma_{ult}-\gamma_{y}\right)+\phi_{0}}.$$

In this model, $\tau_{y}$, $\tau_{ult}$ and $\gamma_{y}$, $\gamma_{ult}$ are the yield and ultimate shear stress, respectively, shear strain.

The parameters that are regarded in the different analytical models for predicting UD compressive strength of FRP are compared in Table 1.

The combined modes model considers the most input parameters and exhibits the best prediction results when compared to experimental results for CFRP [32]. However, the influence of different fibre types in these analytical models has not yet been investigated and the applicability for GFRP is not clear. A compressive test series is carried out for comparison of the models with experimental results for CFRP and GFRP. The necessary plastic shear parameters are determined in tensile tests.

## 3. Experimental Methods

### 3.1. Materials and Sample Preparation

Unidirectional unitex fabrics E-glass *UT-E250* and carbon *UT-C200* from *Gurit Services AG* (Zurich, Switzerland) are used as fibrous reinforcements. The E-glass fabric has a nominal areal weight of $250\;g/m^{2}$ and a fibre tex of 600 for the warp and 10 for the weft direction. The Young’s modulus of the glass fibres is $E_{GF}=80\;G\mathrm{Pa}$. The carbon fabric comprises of 12k rovings with a fibre tex of 800 in warp and 10 in weft direction and an areal weight of $200\;g/m^{2}$. The carbon fibres have a Young’s modulus $E_{CF}=230\;G\mathrm{Pa}$ according to the data sheet. Since the experimental results are compared with prediction models for the compressive strength, a focus during the laminate layup design is set on a similar fibre volume content $\varphi_{f}$ and specimen thickness *t*, especially for the unidirectional specimens for compression tests. To take the difference in density and fibre diameter of glass and carbon fibres into account, it was necessary to use different areal weights for achieving similar values for $\varphi_{f}$ at a constant specimen thickness.

The epoxy resin *Momentive Epikote RIMR 135* (Momentive Performance Materials GmbH, Leverkusen, Germany) with the amine hardener *Momentive Epikure RIMH 134* is used as a matrix system for all specimens. The resin to hardener mixing ratio is 10:3 as recommended by the manufacturer. It is an infusion matrix system with an onset of the glass transition region of $T_{g,onset}=85{}^{\circ}C$ and midpoint glass transition temperature of $T_{g}=93{}^{\circ}C$, according to the data sheet. The matrix Young’s modulus is $E_{m}=2.7\;G\mathrm{Pa}$ according to the data sheet and the shear modulus is $G_{m}=1.0\;G\mathrm{Pa}$ (ν=0.3).

Laminate plates are produced by using vacuum assisted resin transfer moulding (VARTM). The mould is prepared with release agent before the dry fabrics are cut to size and placed inside the mould according to the stacking sequences ${\left[0\right]}_{n}$ for compression and ${\left[+45/-45\right]}_{2s}$ for tensile tests (refer to Table 2). The matrix system is mixed, degassed under vacuum for 15 min and infused via a vacuum and an applied pressure of 2 bar. Curing is done for 20 h at ambient temperature and for 15 h at 80°C in an oven, as recommended for this matrix system. This way, laminate plates with dimensions of length × width ×t=580 mm×280 mm×2 mm are produced. The fibre volume fraction after curing of each laminate is determined via a chemical etching process according to the standard DIN EN 2564 [34]. For the unidirectional specimens, a fibre volume content of $\varphi_{f}=43.5\%$ is measured for both GFRP and CFRP laminates allowing good comparability (refer to Table 2). Therefore, the influence of $\varphi_{f}$ on the evaluation of fibre material with regard to the applicability of the analytical models for predicting the compressive strength is eliminated.

Specimens for compression and tensile tests are cut from the respective plates with a diamond saw. Specimen dimensions for compression tests are according to ASTM-D6641/ASTM-D3410 [35,36] with $l_{c}\times w_{c}=145\;\mathrm{mm}\times 25\;\mathrm{mm}$. Before testing, 2 mm thick GFRP end tabs are applied on the specimens leaving a gauge length of 25 mm between the tabs. Specimen dimensions for tensile tests are set to $l_{t}\times w_{t}=255\;\mathrm{mm}\times 25\;\mathrm{mm}$ according to ASTM D3518 [37]. End tabs consisting of 2 mm thick GFRP/Aluminum stripes are applied on the specimens, resulting in a gauge length of 150 mm. All end tabs were applied with 2-component epoxy adhesive (UHU Endfest-300). Ten specimens of each configuration are tested in the respective test series to take statistical variations from the manufacturing process into account.

### 3.2. Quasi-Static Tests

Quasi static tensile tests of the ±45° specimens are carried out according to ASTM D3518 [37] in a Zwick/Roell Z100 universal test machine (ZwickRoell GmbH, Ulm, Germany) at a cross-head speed of 2mm/min. The load is continuously measured with a load cell with a maximum load of 100 kN. The displacement is recorded with a long-travel extensometer on the specimen surface (measuring distance 50 mm). Transverse strain is calculated with the Poisson’s ratio determined previously to $\nu_{CFRP}=0.75$ and $\nu_{GFRP}=0.70$ for a ±45 layup of CFRP and GFRP, respectively. It has to be noted that this type of strain measurement deviates from the standard, but the calculated results are expected to be of adequate accuracy. The in-plane shear stress is calculated directly from the measured axial load with τ=F/2A [37]. The shear strain is calculated from the longitudinal and the transverse normal strain by using the measured displacement and the Poisson’s ratio.

**Figure 1:**
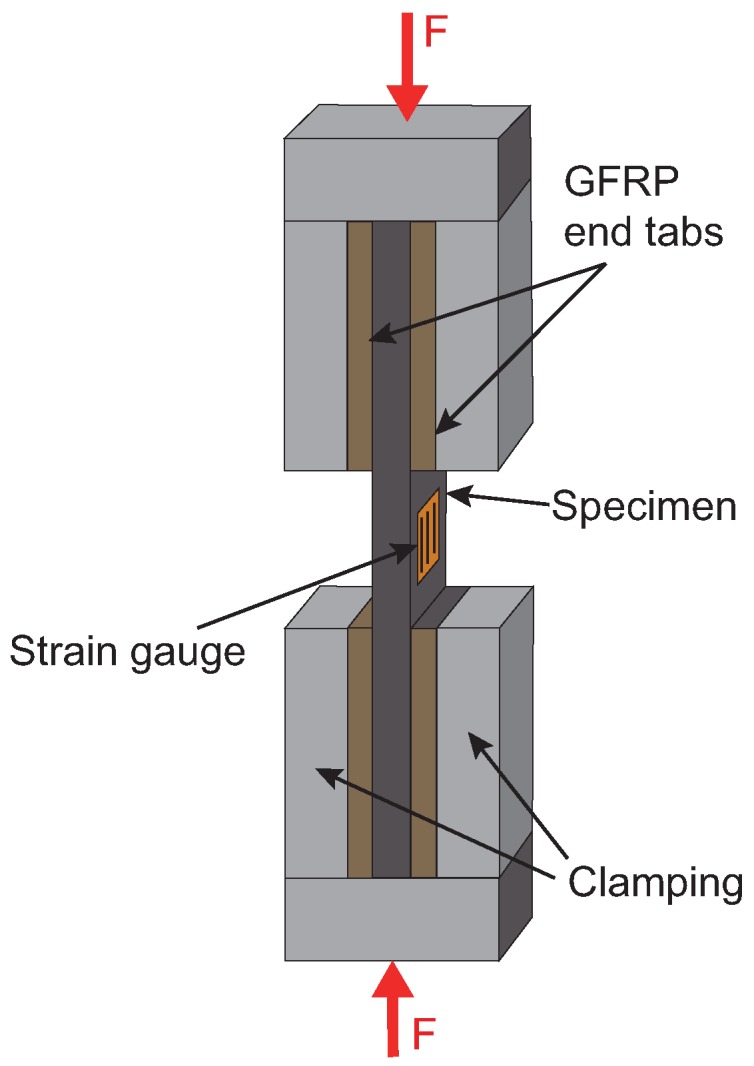
Scheme of the test set-up for compression tests.

Compression tests are executed by using a Zwick-Roell Z400 testing machine with hydraulic clamps. The specimens are mounted in a hydraulic composite compression fixture (HCCF) and mainly loaded by compressive force on their end surfaces. A scheme of the test set-up is shown in Figure 1. The cross-head speed is set to 0.5mm/min. The compressive load is measured by a 400 kN load cell, whereas displacement is measured via the traverse of the machine. The deformation and force are continuously recorded to determine elongation, compressive strength and the modulus of elasticity. Strain gauges are fixed on some specimens of each type to verify that no global bending or out of plane buckling occurs.

### 3.3. Measurement of Fibre Misalignment

Measurement of fibre radii and fibre misalignment, with the method proposed by Yurgartis [38], is carried out by using light microscopy of polished laminate cross sections in an optical microscope (Olympus BX51, Olympus, Hamburg, Germany). Fibre radii of $r_{f,CFRP}=2.87\;\mathrm{mm}\pm 0.27\;\mathrm{mm}$ and $r_{f,GFRP}=6.04\;\mathrm{mm}\pm 0.89\;\mathrm{mm}$ are measured for CFRP and GFRP, respectively. Since the glass fibres are larger in diameter than the carbon fibres, they are expected to be less prone for local microbuckling at small defects [29]. The higher deviation of the glass fibres from the mean diameter value makes the compressive strength of GFRP more difficult to predict.

Several micrograph sections are analysed for each material to determine the in plane fibre misalignment $\phi_{0,IP}$ present in the specimens. Sections were cut under an angle of $\phi_{PC}=10{}^{\circ}$ with regard to 0°-fibre direction. For each micrograph, 200 ellipses are measured and the untransformed fibre orientation ω is determined according to Equation (8) from the ratio of the semi-minor axis *d* to the semi-major axis *l* for each fibre [38]:

(8) $$\sin\left(\omega_{i}\right)=\frac{d_{i}}{l_{i}}.$$

In this equation, ω is the angle with regard to the sectioning plane. A transformation is thus necessary to obtain the misalignment angle ϕ with respect to the zero degree fibre direction. The transformation is calculated with Equation (9) [38]:

(9) $$\phi =\omega -\phi_{PC}.$$

The angle $\phi_{PC}$, referenced to the 0°-direction, is not known with good accuracy, but can be determined from the distribution of the untransformed fibre orientation f(ω). Since the zero degree direction is the mean of the fibre misalignment distribution, $\phi_{PC}$ is equal to the mean of f(ω) and the distribution of the fibre misalignment angle can be calculated with $f\left(\phi \right)=f\left(\omega -\bar{\omega }\right)$ [38].

The out-of-plane (OOP) fibre misalignment $\phi_{0,OOP}$ is determined from micrograph cut sections at the side of the specimens parallel to the fibre direction. OOP misalignment is present because of the nature of the used fabric. The maximum misalignment angles at the crossing points of the rovings with the thermoplastic binding yarns are measured by using the software of the light microscope.

## 4. Results and Discussion

Measurement of in-plane fibre misalignment results in a Gaussian distribution of the untransformed fibre orientation f(ω) and after the transformation of the fibre misalignment angles f(ϕ). The distribution range of f(ϕ) for GFRP is from −4° to +5°. Since 90% of all values are between −2.75° and +2.75°, a mean misalignment angle of $\phi_{0,IP}^{GFRP}=\pm 3^{{}^{\circ}}$ is valid for GFRP. For CFRP, the distribution range is smaller (−2° to +3°) with 92% of all values lying between −2° and +2°. Hence, a mean misalignment angle of $\phi_{0,IP}^{CFRP}=\pm 2^{{}^{\circ}}$ is present in the CFRP specimens. The median misalignment angle as well as the standard deviation of the misalignment for CFRP is smaller compared to GFRP.

The maximum local OOP fibre misalignment is measured to $\phi_{0,OOP}^{GFRP}=2^{{}^{\circ}}$ for GFRP and $\phi_{0,OOP}^{CFRP}\;=\;3^{{}^{\circ}}$ for CFRP. This type of misalignment is assumed to be more critical because the fibres are deflected with regard to loading direction in the plane where kink-band initiation and growth occurs. It is thus critical for damage initiation and growth leading to global buckling under compression loading.

### 4.1. Tensile Tests with Angle-Ply Laminates

**Figure 2:**
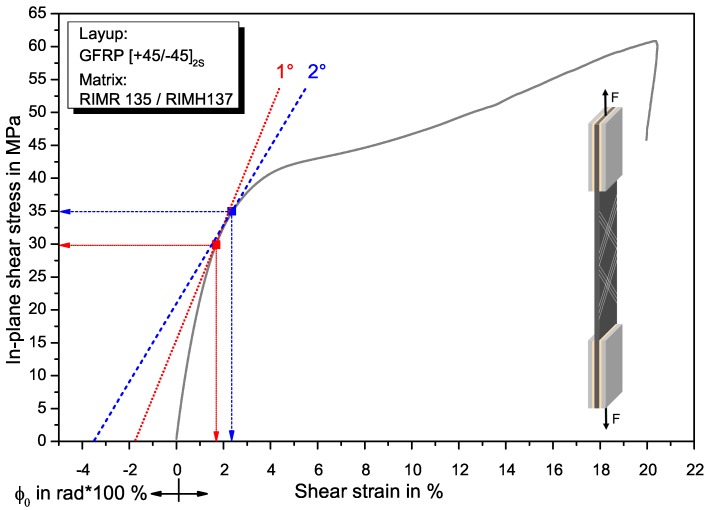
Representative stress–strain diagram with approach for determining the plastic shear yield stress and -strain in tensile tests with a ${\left[\pm 45\right]}_{s}$ layup (example for GFRP).

Figure 2 shows a representative in-plane shear stress versus shear strain curve for GFRP from tensile tests with [+45/−45]${}_{2s}$ specimens. In the diagram, the approach for determining the shear yield parameters for the analytical models from the stress–strain curve is presented. The approach for CFRP is similar. The fibre misalignment angle $\phi_{0}$ in radian is plotted as a negative value on the *x*-axis. From this point, a tangent to the stress–strain curve is constructed. At the contact point of tangent and curve, the yield stress and strain for this angle can be read at the respective axis [30,31,32,33]. In the diagram, this is shown exemplarily for misalignment angles of 1° and 2°. The obtained shear yield stress $\tau_{y}$ and shear yield strain $\gamma_{y}$ for both materials as a function of different fibre misalignment angles $\phi_{0}$ are summarised in Table 3. The shear properties as determined with the tensile tests are summarised in Table 4. The shear modulus $G_{c}$ is determined within the linear slope of the stress–strain curve between 0.01% and 0.05% strain. The shear strength, shear modulus and maximum shear strain for CFRP is higher compared to GFRP (n=10 specimens in each case). Since the same matrix system is used, the difference in shear properties between GFRP and CFRP can be attributed to the fibre type, with the stiffer and stronger carbon fibres accounting for the higher values.

### 4.2. Compression Tests and Comparison with Analytical Models

**Figure 3:**
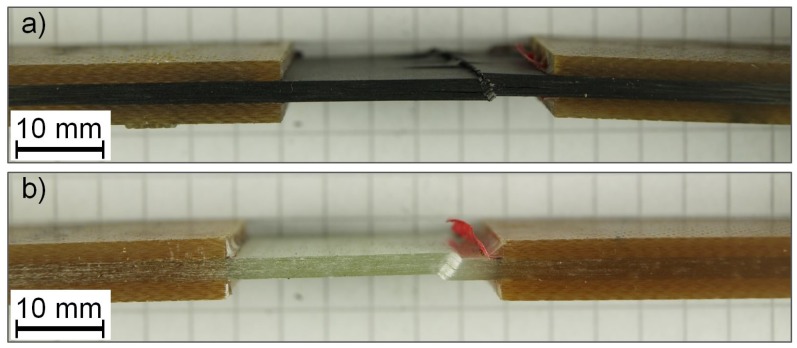
Representative specimens after final failure: (**a**) CFRP; (**b**) GFRP.

The UD compressive strength as experimentally determined is $\sigma_{c,GFRP}=379.6\;M\mathrm{Pa}\pm 17.4\;M\mathrm{Pa}$ for GFRP and $\sigma_{c,CFRP}=569.1\;M\mathrm{Pa}\pm 242\;M\mathrm{Pa}$ for CFRP (refer to Table 4). Compressive strength of CFRP is approx. 50% higher than that of GFRP. The higher strength for CFRP can be attributed to the higher strength of carbon fibres in comparison to glass fibres. Figure 3 shows photos of a representative CFRP and GFRP specimen after final failure. Final failure occurs in the form of a kink-band that is visible for both materials. The CFRP specimens exhibit a slightly higher amount of delaminations and fibre breakage next to the kink-band, which indicates the higher load at failure that results in more severe visible damage.

The prediction accuracy for UD compressive strength of the analytical models is compared with the compression test results. With the determined plastic shear parameters (Table 3) and the material properties of fibre and matrix (refer to Section 3.1), the strength is calculated for different fibre misalignments $\phi_{0}$ by using the various models presented briefly in Section 1.

The predicted strength values of the different models are summarised in Table 5 and compared with the experimental results. A fibre misalignment angle of 3° is selected for this comparison because it reflects the measured misalignment in the specimens well. Best prediction accuracy is achieved with the kinking model from Budiansky [19,20], especially for CFRP.

The **shear model** from Rosen [2] (refer to Equation (1)) highly overestimates the compressive strength with a predicted strength of approximately 1785 MPa for both GFRP and CFRP in the shear mode. For the extension mode, the values are even higher. The model is not able to differentiate between different fibre types. This was expected because only the matrix shear modulus and the fibre volume fraction are used as input parameters in this approach to predict the strength of a material with a very complex damage process under the given load case and is in accordance with previous results from other authors [32].

**Figure 4:**
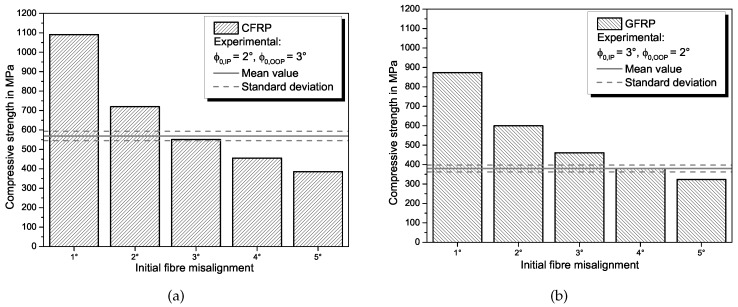
Comparison of prediction from the fibre kinking model from Budiansky [19,20] for different misalignment angles with experimental results for UD compressive strength of CFRP (**a**) and GFRP (**b**).

For the **fibre kinking model** from Budiansky [19,20], the compressive strength is calculated with Equation (2). The model predictions in comparison with the experimental results are shown in Figure 4 for GFRP and CFRP. For small misalignment angles, the model highly over-predicts the compressive strength. The predicted compressive strength of 550.7 MPa for CFRP with a fibre misalignment angle of 3° correlates well with the experimental results, with the predicted value being within the standard deviation but below the mean value. As the fibre type is regarded only indirectly via the results from the shear test in the model parameters, the difference between predicted strength and failure strength is higher for GFRP. For a misalignment angle of 4°, which is higher than the measured misalignment in the GFRP specimens, the predicted value of 378.5 MPa is within the standard deviation of the experimentally determined strength. The better agreement of predicted values with experimental results compared to the literature [16,17,32] can be explained with the fibre volume content $V_{f}$ that is lower in our specimens. With a decreasing fibre volume content, plastic kinking is facilitated and the matrix properties become more and more relevant. Therefore, the fibre kinking model, which is mainly based on the matrix shear behaviour, is more accurate for lower $V_{f}$.

In the **fibre microbuckling model** from Berbinau et al. [16], the fibre type determines the fibre cross section area $A_{f}$ and second modulus of inertia *I* in Equation (4). For the calculation of $I=\pi \;\cdot \;(r_{f}^{4}/4)$ and $A_{f}=\pi \;\cdot \;r_{f}^{2}$, the measured mean fibre radius is used. The amplitude value of the unstressed fibre $V_{0}$ is calculated with Equation (10). It is reported that the wavelength λ equals the kink band width [39] and for $\lambda_{0}$ a value of $\lambda_{0}=10\;d_{f}$ is reasonable [9,13,14,16,17], thus this approach is used here as well:

(10) $$V_{0}=\left(\frac{\lambda_{0}}{\pi }\right)\;\cdot \;\tan\left(\phi_{0}\right).$$

A graphic representation of $V/V_{0}$ is plotted over the compressive stress and an asymptotic increase of the fibre amplitude predicts a compressive strength of approximately 720 MPa for CFRP. Therefore, this model overestimates the compressive strength, which is critical for conservative design of composite parts and in contrast to what is previously reported for this model [32]. The predicted strength for our material is lower compared to the values predicted for CFRP with a higher volume fraction [17]. Hence, the general influence of fibre content is represented qualitatively correct by the model, but the predicted value has a large error compared to the experiments with specimens that have a lower $V_{f}$. Decreasing $V_{f}$ leads to a higher decrease of $\sigma_{c}$ than predicted by the model.

For GFRP, the microbuckling model in the current form is not applicable because the graphic representation of $V/V_{0}$ over the compressive stress results in an asymptotic decrease. With the slope of $V/V_{0}$ tending to zero instead of to infinite as expected, the compressive strength cannot be read at the *x*-axis and an adaption of the microbuckling model is necessary to be applicable for GFRP. This is related to the fact that the larger fibre diameter of the glass fibres that determines the moment of inertia *I* and the cross section area $A_{f}$, in combination with the lower fibre Young’s modulus $E_{f}$, in comparison to CFRP, leads to a negative denominator in Equation (4) and thus a decrease of $V/V_{0}$ over $\sigma_{c}$. In other words, the term $x_{2}=A_{f}\;\cdot \;G_{c}$ has a higher value than the term $x_{1}=E_{f}\;\cdot \;I\;\cdot \;{\left(\pi /\lambda \right)}^{2}$ for glass fibres. Consequently, the fibre type and diameter $r_{f}$, are important factors and should be considered for compressive strength prediction. When writing Equation (4) with the introduced abbreviations in the form $\frac{V}{V_{0}}={\left(1-\frac{P}{x_{1}-x_{2}}\right)}^{-1}$, for an asymptotic increase, $x_{1}>x_{2}$ must be valid, which is not the case for GFRP. This can be avoided, when the absolute value of the term in the denominator is used, leading to the adapted Equation (11). This equation with the absolute values for the denominator is applicable for both CFRP and GFRP:

(11) $$\frac{V}{V_{0}}={\left[1-\frac{P}{\left|E_{f}I{\left(\pi /\lambda \right)}^{2}-A_{f}G_{c}\right|}\right]}^{-1}.$$

**Figure 5:**
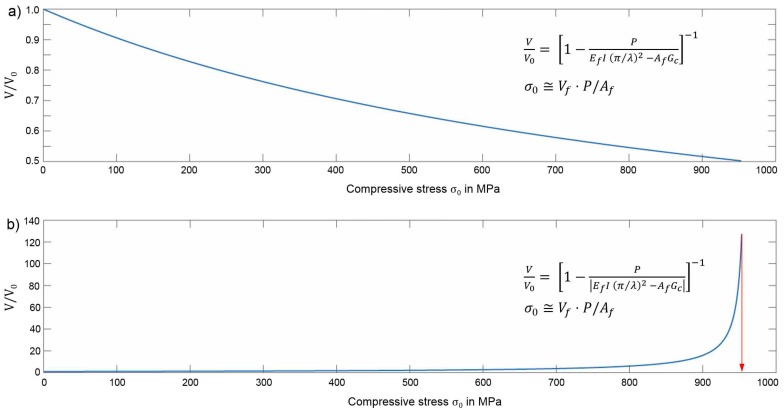
Application of (**a**) the original and (**b**) the adapted microbuckling model for predicting the compressive strength of GFRP dominated by fibre microbuckling.

The graphic representation of $V/V_{0}$ versus compressive stress $\sigma_{0}$ for GFRP is shown in Figure 5 for both the original microbuckling model from Equation (4) in Figure 5a and for the adapted model from Equation (11) in Figure 5b. Curves are plotted for misalignment angles between 1° and 5°, but the influence of fibre misalignment of the graphic representation of $V/V_{0}$ and thus the predicted strength is negligible.

With Equation (11), a compressive strength of 950 MPa is predicted for GFRP (refer to Figure 5). This is higher than the predicted strength for CFRP, which results from the increased stability of thicker fibres against microbuckling in the model but does not represent realistic behaviour. When using comparable specimen geometry, CFRP achieves higher compressive strength than GFRP, as is also the case in the experiments. Regarding the predicted strength values, the adapted microbuckling model significantly overestimates the compressive strength. The prediction error is even larger for GFRP due to the fact that a higher predicted strength coincides with lower measured strength when compared to CFRP. The suggested adaption allows application of the microbuckling model, although originally derived for CFRP. However, a higher predicted strength for GFRP than for CFRP is not reasonable.

The microbuckling model uses the shear modulus of the composite $G_{c}$ for predicting the microbuckling behaviour. For thicker fibres and lower fibre volume fractions, it can be argued that the local microbuckling of a fibre depends more on the shear modulus of the surrounding matrix than on that of the composite due to the larger inter-fibre distance. This could be the case for the GFRP used in this study, which exhibits a significantly lower fibre volume fraction ($V_{f}=43.5\%$) compared to the CFRP prepreg system against which the microbuckling model was verified [16,17]. When using the matrix shear modulus $G_{m}$ instead of $G_{c}$ in Equation (11), a compressive strength of 220 MPa is predicted for GFRP, which is lower than the experimentally determined strength but far more realistic. For CFRP, a compressive strength of 180 MPa is predicted when using the matrix shear modulus instead of the composite shear modulus in Equation (11). This underestimation of strength agrees better with the general behaviour of the microbuckling model as described in literature that led to the introduction of the combined modes model [32].

**Figure 6:**
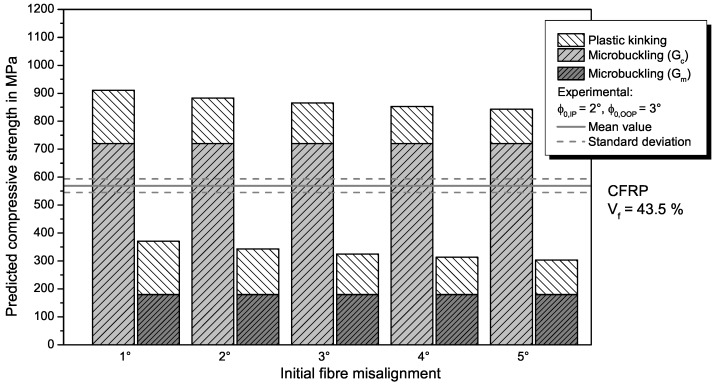
Comparison of prediction from the combined modes model from Jumahat [32] for different misalignment angles with experimental results for UD compressive strength of CFRP. The influence of using either the shear modulus of the composite $G_{c}$ or that of the matrix $G_{m}$ within the model is plotted as well.

**Figure 7:**
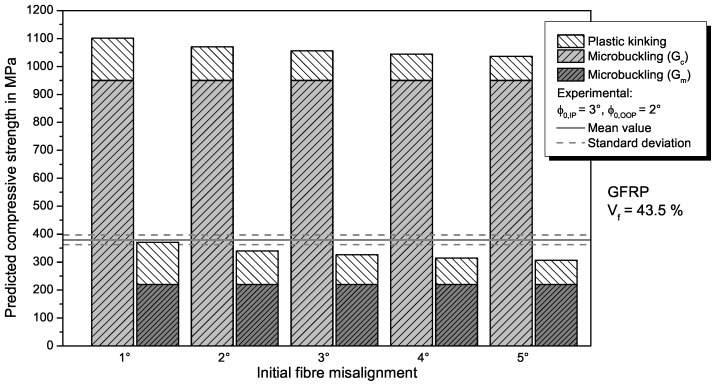
Comparison of prediction from the adapted combined modes model from Jumahat [32] for different misalignment angles with experimental results for UD compressive strength of GFRP. The influence of using either the shear modulus of the composite $G_{c}$ or that of the matrix $G_{m}$ within the model is plotted as well.

The **combined modes model** by Jumahat [32] consists of a microbuckling part and a kinking part, as described by Equation (6). For calculating the ratio of compressive strength attributed to fibre microbuckling, the adapted Equation (11) is used, so that the combined model is also applicable for GFRP. The fibre kinking ratio is calculated with Equation (7), with the parameters determined in the tensile tests for the respective material. Results from the combined modes model for different fibre misalignment angles $\phi_{0}$ in comparison with the experimental results (mean value and standard deviation) are shown in Figure 6 for CFRP and in Figure 7 for GFRP. Realistic misalignment angles between 1° and 5° are chosen to analyse a certain range of fibre misalignment with the model.

Since the microbuckling model already overestimates the compressive strength of the specimens, the combined modes model by Jumahat et al. [32] does so as well. The predicted UD compressive strength of 865 MPa for CFRP with an initial fibre misalignment of 3°, which is the local out-of-plane misalignment measured in our specimens, is lower compared to predicted strength values reported for prepreg-CFRP with a higher $V_{f}$ [32], but significantly higher than measured specimen strength. Therefore, the model is able to represent the general influence of a lower fibre volume fraction with its prediction, but leads to a overestimation of compressive strength for lower $V_{f}$. For GFRP, the combined model also predicts higher compressive strength values for the investigated range of misalignment than measured in the experiments. For a misalignment angle of 3°, the predicted value is 1056 MPa. This is unrealistically higher that the value predicted for CFRP and results from the higher microbuckling ratio compared to CFRP because the microbuckling model predicts higher strength for GFRP.

If the matrix shear modulus $G_{m}$ instead of the composite modulus is used to calculate the microbuckling ratio, the prediction accuracy for GFRP is quite good. The use of the matrix shear modulus is motivated by the lower fibre volume fraction that results in more matrix dominated microbuckling of the fibres. For small misalignment angles, the predicted strength is within the standard deviation of the test results. For larger misalignment angles, the strength is slightly underestimated, which is less critical for conservative design. For CFRP, usage of $G_{m}$ is not meaningful because the compressive strength is highly underestimated.

When comparing the different analytical models for predicting the compressive strength of FRP (refer to Table 5), the fibre kinking model achieves the best results in comparison to the experiments. This is unexpected because it is in contrast to previous investigations [16,17,32]. Probable reasons for this deviation are the material and the manufacturing process. In the other investigations, a CFRP prepreg material with a fibre volume fraction of approximately $V_{f}=65\%$ was used that was autoclave cured. In our study, we used a non-crimp fabric and prepared specimens via a VARTM process, resulting in a lower fibre volume fraction ($V_{f}=43.5\%$). Both the infusion process and the achieved fibre volume fraction are typical for many applications of FRP such as wind turbine blades or sporting goods and thus of relevance for an accurate prediction of compressive properties. As expected, the lower fibre volume fraction results in a lower compressive strength compared to the values in literature [16,17,32]. The trend of decreasing strength with lower $V_{f}$ is represented by the kinking model and the combined modes model, although the latter highly overestimates the strength for lower $V_{f}$. It can be concluded that the matrix properties become more important with decreasing fibre content and that fibre kinking is the dominant failure mechanisms in that case. This is more pronounced for GFRP than for CFRP, where usage of matrix instead of composite shear properties leads to accurate prediction of compressive strength in the combined modes model considering both microbuckling and kinking.

It has to be noted that, in our experiments, the fibres exhibit circular cross sections and such a shape is used for calculation of fibre cross section area $A_{f}$ and moment of inertia *I*. However, in some composite parts, the fibre cross-section is of a kidney shape, which influences the mechanical properties and failure behaviour under compressive loading [40]. This should be considered, when applying the analytical models to predict the UD compressive strength of laminates with kidney-shaped fibres (e.g., by different equations for calculating $A_{f}$ and *I* in the microbuckling and combined modes model).

## 5. Conclusions

Existing models for predicting the UD compressive strength of FRP are compared with experimental results for CFRP and GFRP. A fibre kinking model is the most accurate and for CFRP within the standard deviation compared to the experiments. This is in contrast to previous investigations, where a combined modes model that considers microbuckling as well as plastic kinking achieved the best prediction accuracy. The deviation is explained by the variance in fibre volume fraction. For lower fibre volume fractions as in this study, the matrix properties apparently play a more important role and kinking instead of microbuckling is the dominant failure mechanism. However, in the original form, the combined modes model is not able to predict the compressive strength for GFRP. The model is modified by considering the stiffness to diameter ratio of the fibres. The adapted model is applicable to GFRP, but significantly overestimates the compressive strength of both GFRP (+178%) and CFRP (+52%) for the used fibre volume fraction. The fibre properties, especially the fibre diameter and stiffness, are not adequately considered. Further adaption of the models regarding the fibre morphology and a more detailed evaluation on the influence of fibre volume fraction are still necessary to consider different fibre types such as carbon, glass or natural fibres for the various applications of FRP.

## Figures and Tables

**Table 1:** Comparison of input parameters for different analytical models for compressive strength prediction of FRP UD laminates.

	Rosen [2]	Budiansky [19,20]	Berbinau [16]	Jumahat [32]
Fibre misalignment $\phi_{0}$	X	✓	✓ *	✓
Fibre radius $r_{f}$	X	X	✓	✓
Fibre Young’s modulus $E_{f}$	✓	X	✓	✓
Matrix shear modulus $G_{m}$	✓	X	✓	✓
Shear yield stress $\tau_{y}$	X	✓	X	✓
Shear yield strain $\gamma_{y}$	X	✓	X	✓

* $\phi_{0}$ is considered, but variation has very small influence on prediction results..

**Table 2:** Overview over the used non-crimp fabric fibre materials, measured laminate fibre volume fractions and material parameters.

Fibre Type	Layup	Areal Weight in g/m${}^{2}$	$\varphi_{f}$ in %
Carbon fibre	${\left[0\right]}_{8}$	200	43.49
Carbon fibre	[+45/−45]${}_{2s}$	200	42.51
Glass fibre	${\left[0\right]}_{9}$	250	43.53
Glass fibre	[+45/−45]${}_{2s}$	250	38.86

**Table 3:** Plastic shear yield stress and -strain for different fibre misalignment angles as determined with a graphical method from tensile tests with ${\left[\pm 45\right]}_{s}$ angle-ply laminates.

Material	$\phi_{0}$	$\tau_{y}$ in MP	$\gamma_{y}$ in %
GFRP	1°	29.9	1.68
GFRP	2°	35.0	2.35
GFRP	3°	36.7	2.72
GFRP	4°	38.1	3.08
GFRP	5°	38.9	3.31
CFRP	1°	36.0	1.55
CFRP	2°	41.5	2.27
CFRP	3°	44.6	2.87
CFRP	4°	46.0	3.14
CFRP	5°	47.4	3.57

**Table 4:** Experimental results: Shear properties determined in quasi-static tensile tests with ${\left[\pm 45\right]}_{s}$ laminates and compressive strength of GFRP and CFRP (n=10 specimens).

Material	Shear Strength $\tau_{\mathrm{ult}}$	Shear Modulus $G_{c}$	Max. Shear Strain $\gamma_{\mathrm{ult}}$	Compressive Strength $\sigma_{c}$
GFRP	(61.4±1.6) MPa	(2.70±0.12) GPa	(20.8±1.4)%	(379.6±17.4) MPa
CFRP	(85.5±2.1) MPa	(3.07±0.06) GPa	(25.8±2.2)%	(569.1±24.2) MPa

**Table 5:** Comparison of predicted UD compressive strength for different analytical models with experimental values for CFRP and GFRP for a fibre misalignment angle of 3°.

	Experiment	Rosen [2]	Budiansky [19,20]	Berbinau [16]	Jumahat [32]
$\sigma_{c}$ CFRP in MPa	569.1±24.2	1785	551	720	865
Deviation CFRP in MPa	-	1216	−18 *	151	296
Deviation CFRP in %	-	214	−3 *	27	52
$\sigma_{c}$ GFRP in MPa	379.6±17.4	1785	461	950	1056
Deviation GFRP in MPa	-	1405	81	570	676
Deviation GFRP in %	-	370	21	150	178

* Deviation is smaller than the standard deviation of test results.

## Abbreviations

The following abbreviations are used in this manuscript:

CFRP	Carbon fibre reinforced polymer
FRP	Fibre reinforced polymer
GFRP	Glass fibre reinforced polymer
IP	In-plane
OOP	Out-of-plane
UD	Unidirectional
VARTM	Vacuum assisted resin transfer moulding
